# Differential regulation of the c-Myc/Lin28 axis discriminates subclasses of rearranged MLL leukemia

**DOI:** 10.18632/oncotarget.8199

**Published:** 2016-03-19

**Authors:** Lili Chen, Yuqing Sun, Jingya Wang, Hui Jiang, Andrew G. Muntean

**Affiliations:** ^1^ Department of Pathology, University of Michigan Medical School, Ann Arbor, MI, USA; ^2^ Department of Biostatistics, School of Public Health, University of Michigan, Ann Arbor, MI, USA

**Keywords:** acute myeloid leukemia, MLL, c-Myc, Lin28, let-7

## Abstract

*MLL* rearrangements occur in myeloid and lymphoid leukemias and are generally associated with a poor prognosis, however this varies depending on the fusion partner. We modeled acute myeloid leukemia (AML) in mice using various MLL fusion proteins (MLL-FPs) and observed significantly different survival outcomes. To better understand the differences between these leukemias, we examined the genome wide expression profiles of leukemic cells transformed with different MLL-FPs. RNA-sequencing and pathway analysis identified the c-Myc transcriptional program as one of the top distinguishing features. c-Myc protein levels were highly correlative with AML disease latency in mice. Functionally, overexpression of c-Myc resulted in a more aggressive proliferation rate in MLL-FP cell lines. While all MLL-FP transformed cells displayed sensitivity to BET inhibitors, high c-Myc expressing cells showed greater resistance to Brd4 inhibition. The Myc target *Lin28B* was also differentially expressed in MLL-FP cell lines in agreement with *c-Myc* expression. Examination of Lin28B miRNAs targets revealed that *let-7g* was significantly increased in leukemic cells associated with the longest disease latency and forced *let-7g* expression induced differentiation of leukemic blasts. Thus, differential regulation of the c-Myc/Lin28/*let-7g* program by different MLL-FPs is functionally related to disease latency and BET inhibitor resistance in *MLL* leukemias.

## INTRODUCTION

Rearrangements at chromosome 11q23 (*MLL* translocations) produce fusion proteins (MLL-FPs) composed of the N-terminus of Mixed Lineage Leukemia (MLL) and various c-terminal fusion partners. *MLL* translocations are associated with approximately 10% of human acute leukemia, including pediatric, adult and therapy-related leukemia [1]. However, this chromosomal aberration is extremely common in infant leukemia patients, with ~70% of infant Acute Lymphoid Leukemia (ALL) and ~30% of infant Acute Myeloid Leukemia (AML) patients harboring MLL translocations [1]. Patients harboring *MLL* translocations have a relatively poor prognosis, however this can vary according to the fusion partner. For example, favorable outcomes with a 5-year event free survival (EFS) rate of 92% were observed in pediatric AML patients with t(1:11)(q21;p23) (MLL-AF1q) while poor clinical outcomes were observed for t(6;11)(q27;q23) (MLL-AF6), t(10;11)(p11.2;q23) (MLL-ABI1) and t(4;11)(q21;q23) (MLL-AF4) with EFS rates of 11%, 17% and 29% respectively [2, 3]. These data suggest different MLL fusion proteins may utilize alternative mechanisms of transformation to activate unique gene programs that lead to more aggressive diseases. Indeed, recent biochemical characterization of MLL fusion proteins has revealed differing mechanisms of transformation dependent on the fusion partner.

Since its discovery [4], more than 100 MLL fusion partners have been identified. MLL translocations result in the loss of the MLL c-terminus containing a SET domain responsible for H3K4 methyltransferase activity and gene activation [5, 6]. Although the functions of all resultant fusion proteins have not been fully characterized, some commonalities exist, such as the up-regulation of downstream genes, *Hoxa9* and *Meis1*, which in combination can induce aggressive leukemia in recipient mice [7, 8]. MLL fusion partners can be classified by their subcellular localization, nuclear and cytoplasmic. Recent seminal work by a number of groups has revealed that several of the most common nuclear translocation partner genes (including *AF9, ENL, AFF1 (AF4), AFF4 (AF5q31), AF10, AF17* and *ELL*) assemble into a transcriptional activation complex that includes p-TEFb and/or the histone H3K79 methyltransferase DOT1l. Translocation of *MLL* with members of this complex results in deregulated transcriptional activation of target genes due to deregulated phosphorylation of RNA pol II CTD by p-TEFb and increased H3K79 methylation by DOT1L [9-14]. Therapeutic targeting of Dot1L enzymatic activity has provided a potential therapy strategy for MLL leukemia [15].

Conversely, structure/function studies of MLL-FPs bearing cytoplasmic partners, such as AF1p and GAS7, have revealed that coiled-coil oligomerization motifs found in cytoplasmic partners are necessary for transformation [16, 17]. This has also been demonstrated for MLL-AF6 where RA motifs in AF6 were necessary and sufficient for MLL-FP transformation [18]. Interestingly, MLL-AF6 leukemias remain sensitive to DOT1l inhibitors [19]. Another important distinction between cytoplasmic and nuclear fusions is the dependence on Hoxa9. The MLL-AF9 fusion protein, which interacts with p-TEFb/DOT1l, is not capable of inducing leukemia in recipient mice when introduced into *Hoxa9* deficient cells [20], while the cytoplasmic fusion MLL-GAS7 is capable of driving leukemia in cells deficient for either *Hoxa9* or *Hoxa7* [21]. These data point to differences in gene program induction by different MLL-FPs. Indeed, comparisons of primary leukemic cells derived from different MLL fusion proteins have revealed differences in c-kit surface expression that may account for differences in disease latency [22]. Still the gene programs induced directly by different MLL fusion proteins remain unclear.

We modeled MLL leukemia in mice to gain a better understanding of the gene programs induced by various nuclear and cytoplasmic MLL-FPs. Our mouse models of MLL rearranged leukemia display differences in disease latency reflecting differences seen amongst patients with MLL rearrangements. Using genome-wide expression and pathway analysis, we discovered differential activation of the c-Myc transcriptional pathway as a distinguishing feature of MLL leukemia with a short and long disease latency. Indeed, modulation of c-Myc by overexpression or chemical inhibition establishes c-Myc as both universally required in MLL leukemia and determinant of cell proliferation rates. We also observe differential regulation of the c-Myc target and miRNA regulator Lin28B amongst various MLL-FPs. Negative regulation of miR-150 by Lin28B was observed in all MLL-FP cell lines, which is necessarily downregulated in 11q23 leukemias. Interestingly, weaker repression of another Lin28B target, *let-7g*, was correlated with c-Myc expression levels and proliferation rates observed in MLL cell lines and promoted a differentiation phenotype in all MLL-FP cells. These data suggest different 11q23 rearrangements differentially activate the *Myc* transcriptional program that, in part and with exception, contributes to different disease latencies.

## RESULTS

### Disease latency varies with fusion partner in a mouse model of MLL leukemia

**Figure 1:**
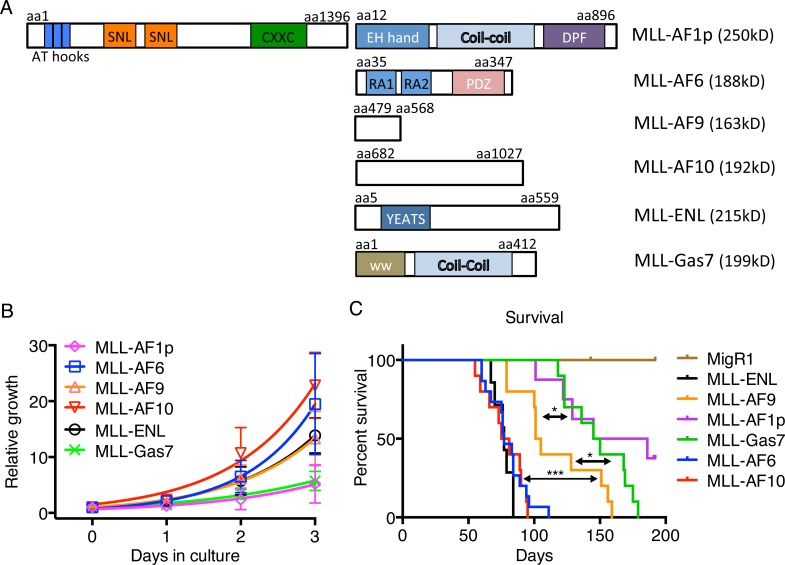
MLL fusion partners are associated with different phenotypes **A.** Schematic of MLL-FPs used in this study with structural domains indicated. Predicted molecular weights are shown. Amino acid location is indicated. **B.** Proliferation of MLL-FP leukemia cell lines. Error bars indicate standard deviation from two independent experiments with technical duplicates. **C.** Survival curve of mice transplanted with indicated MLL-FP transduced cells. MLL-AF1p: *n* = 8; MLL-AF6, *n* = 15; MLL-AF9, *n* = 10; MLL-AF10: *n* = 10; MLL-ENL: *n* = 7; MLL-Gas7: *n* = 10. ***, *p* < 0.001 *, *p* < 0.05 (determined by Log-rank test)

To better understand the direct roles of different MLL fusion proteins on transformation, cell lines were generated by transducing Kit^+^ enriched mouse hematopoietic stem and progenitor cells with retroviruses encoding MLL-AF1p (distinct from the MLL-AF1q fusion reported in Balgobind et al. [2]), MLL-AF6, MLL-AF9, MLL-AF10, MLL-ENL and MLL-Gas7 (Figure 1A). To gain insight into the proliferative capacity of these cell lines, we performed an *in vitro* growth assay in the presence of Il3. MLL-AF6, MLL-AF9, MLL-AF10 and MLL-ENL cells grew significantly faster than MLL-AF1p or MLL-Gas7 cells suggesting fusion protein intrinsic differences in transformation (Figure 1B). Expression of MLL-FP proteins in mouse leukemic cell lines was confirmed by qPCR and western blotting (Supplemental Figure 1A). While MLL-FP expression varied, this did not correlate with the growth rate suggesting differences in proliferation are not correlated to MLL-FP protein level. While all MLL-FP leukemic cell lines displayed both c-Kit^hi^ and c-Kit^low^ populations, MLL-AF1p, MLL-AF6, MLL-AF10 and MLL-Gas7 cells showed distinct populations of c-Kit^hi^ and c-Kit^low^ in contrast to MLL-AF9 and MLL-ENL cells (Supplemental Figure 1B). All MLL-FP cell lines displayed Cd11b surface expression on almost all cells consistent with a myeloid leukemia phenotype. Together, only minor differences in cell surface expression of c-Kit and Cd11b were observed between MLL-FP cell lines that poorly correlated with cellular proliferation.

To investigate how MLL fusion partners influence disease latency, we performed *in vivo* leukemogenesis assays using syngeneic bone marrow transduction and transplantation. Lin-c-kit^+^ bone marrow cells were harvested from C57BL/6 mice, transduced with MLL-FPs, transplanted into lethally irradiated recipient mice and monitored for disease. Interestingly, disease latency varied with MLL fusion partner (Figure 1C). Most recipients receiving MLL-AF6, MLL-AF10 and MLL-ENL cells developed leukemia in 2 to 3 months post transplantation. The median survival for MLL-AF6, MLL-AF10 and MLL-ENL is 77, 78, 77 days respectively. MLL-AF9 mice developed leukemia with a significantly longer median survival of 103 days. MLL-AF1p and MLL-Gas7 mice displayed a significantly longer disease latency compared to MLL-AF9 with a median latency of 165.5 and 147.5 days respectively (Figure 1C). The short disease latency of MLL-AF6 and MLL-AF10 mice is consistent with aggressive disease in human patients associated with t(6;11) or t(10; 11) translocations. Further, the differences in disease latency in our mouse model are reflective of variable disease latencies seen in patients harboring different MLL translocations [2, 3]. MLL-AF1p and MLL-Gas7 mice had significantly longer disease latency and some MLL-AF1p mice did not develop leukemia during our disease-monitoring period. It should be noted that the most aggressive fusion clinically, MLL-AF6, displayed a similar latency as the standard risk MLL-ENL which may reflect differences between mice and humans. All mice displayed splenomegaly at their moribund state (Supplemental Figure 1C). Histological staining showed increased blast cells in MLL-FP mouse peripheral blood, disrupted spleen structures and infiltration of blast cells into spleen and liver (Supplemental Figure 1D). Bone marrow cytospins confirmed the presence of immature myeloid cells with high nuclear to cytoplasm ratios consistent with the development of a myeloid leukemia (Supplemental Figure 1D).

### MLL-FP initiates distinguishable transcriptional programs during leukemic transformation

**Figure 2:**
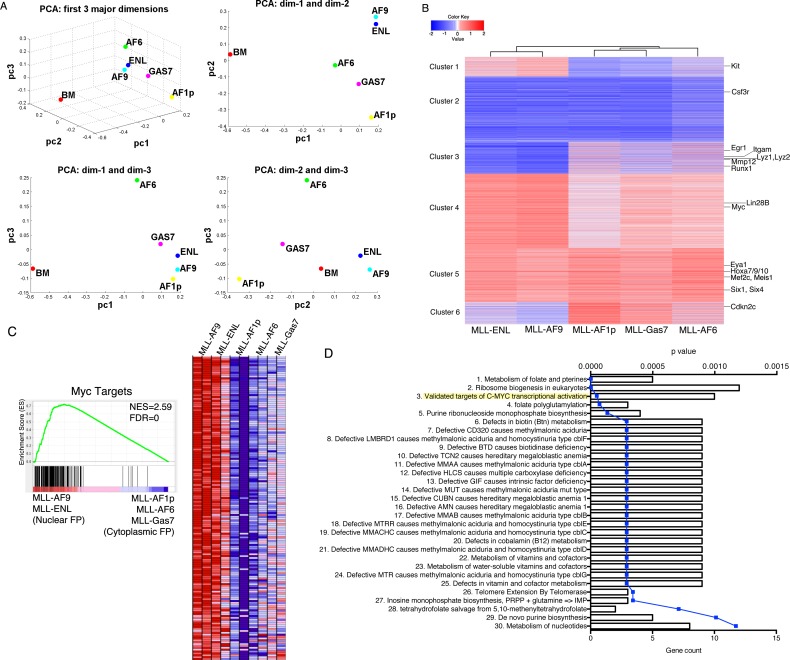
MLL-FPs induce distinguishable transcriptional programs **A.** 3D-principle component analysis (PCA) of averaged counts per million (CPM) data from RNA collected from duplicate samples of MLL-FP cells. 2D projections for each dimension are shown. **B.** Unsupervised clustering of averaged CPM data of all genes expressed in at least one sample. Heat map shows expression pattern in relation to lin^−^c-kit^+^ bone marrow cells. MLL-FP target genes and hematopoietic differentiation genes are listed. Six distinct clusters of genes are denoted. **C.** Enrichment of Myc targets was detected in the nuclear fusion group (MLL-AF9 and MLL-ENL) compared to the cytoplasmic fusion group (MLL-AF-1p, MLL-AF6 and MLL-GAS7) as determined by GSEA. Heatmap shows expression of core genes that contribute to pathway enrichment. Red indicates high expression. Blue indicates low expression. NES: normalized enrichment score. FDR: false discovery rate. **D.** Gene enrichment analysis performed by Toppgene. Averaged CPMs from MLL-AF9 and MLL-ENL were divided from averaged CPMs from MLL-AF1p, MLL-GAS7 and MLL-AF6 and genes with > 1.5-fold change ware analyzed. Results from cluster 4 analysis are shown. Bars indicate gene count, blue line indicates p value.

We next asked whether distinct MLL-FPs initiate distinguishable gene programs based on fusion partner. Some programs, such as upregulation of the *HoxA* cluster genes and Meis1 to block differentiation or *Myb/Hmgb3/Cbx5* utilized for maintaining leukemic stem cells are activated independent of fusion partner [22, 23]. However, differences in disease latency and mechanisms of transformation suggest unique programs may be initiated. To test this RNA sequencing was performed on RNA extracted from two independently derived cell lines transformed with different MLL-FPs. Non-transformed lin- kit+ bone marrow cells were used as control in comparison to MLL-AF9, MLL-ENL, MLL-AF6, MLL-AF1p and MLL-GAS7. Principle component analysis indicated that MLL-leukemia cells initiated transcriptional programs distinct from lin- kit^+^ bone marrow cells (Figure 2A). MLL-AF9 and MLL-ENL grouped with similar gene programs consistent with these two MLL-FPs transforming through enhanced transcriptional elongation due to association with p-TEFb and/or DOT1l and highly homologous sequences [24]. Conversely, MLL-AF6, MLL-AF1p and MLL-Gas7 programs are distinct from both MLL-AF9 and MLL-ENL and from each other suggesting gene programs initiated by MLL-FPs with dimerization motifs are unique (Figure 2A). Next we performed unsupervised hierarchical clustering of MLL-FP cell lines and K-means clustering of genes visualized by comparison to expression in lin- kit^+^ bone marrow cells (Figure 2B). These data similarly suggest that MLL-AF9 and MLL-ENL initiate similar transcriptional programs that are distinct from MLL-AF1p, MLL-Gas7 and MLL-AF6 (Figure 2B). We further noted that while several sets of genes were similarly regulated by all MLL-FPs (clusters 2 and 5), differential regulation of some gene sets occurred between nuclear and cytoplasmic MLL-FPs (clusters 1, 3, 4 and 6) (Figure 2B, Supplemental Table 1). To identify the biological pathways that distinguish the nuclear fusion group (MLL-AF9 and MLL-ENL) and the cytoplasmic fusion group (MLL-AF6, MLL-AF1p and MLL-GAS7), we performed Gene Set Enrichment Analysis (GSEA). Interestingly, Myc targets were detected as the top gene sets enriched in MLL-AF9 and MLL-ENL cell lines compared to MLL-AF1p, MLL-AF6 and MLL-GAS7 (Figure 2C). In addition, we performed pathway analyses on gene clusters showing differential expression in Figure 2B. Expression values for the nuclear fusion group (MLL-AF9 and MLL-ENL) and cytoplasmic group (MLL-AF1p, MLL-GAS7, MLL-AF6) were averaged retrospectively, and compared between the two groups. Genes with ≥1.5 fold change were analyzed using Toppfun [25]. Targets of c-myc transcriptional activation and genes containing a Myc/Max binding sequence were significantly enriched in the cluster 4 nuclear fusion group compared to the cytoplasmic fusion group (Figure 2D, Supplemental Figure 2A). Further, the log2 fold change of Myc target genes (defined in Figure 2D and Supplemental Table 2) in MLL-FP cells compared to Kit^+^ bone marrow cells shows a significantly higher expression in the nuclear group compared to the cytoplasmic group (Supplemental Figure 2B). These data suggest that the Myc transcriptional program is differentially regulated by MLL-FPs. Importantly, previously reported direct targets of MLL-FPs, such as *Hoxa7, Hoxa9, Hoxa10, Meis1, Eya1, Six4, Mef2c* and others were up-regulated uniformly in all MLL-FP leukemias compared to bone marrow (Figure 2B (cluster 5), Supplemental Figure 2C), suggesting these do not account for differences in disease latency reported above (Figure 1C) [26, 27].

### Higher c-Myc expression imparts higher proliferative capacity and resistance to Brd4 inhibition

**Figure 3:**
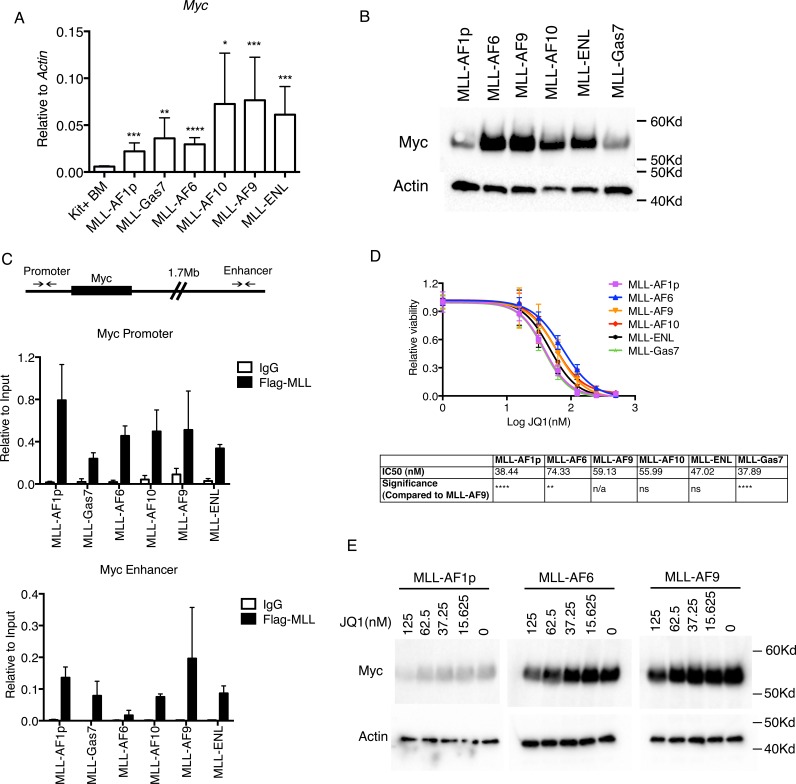
*c-Myc* expression distinguishes MLL-FP leukemias **A.** qPCR detecting the expression of *c-Myc* transcripts. Error bars indicate standard deviation of ≥ 3 independent experiments. Statistics were done by *t*-test with each MLL-FP cells compared to Kit+ BM. *, *p* < 0.05; **, *p* < 0.01; ***, *p* < 0.001; ****, *p* < 0.0001 **B.** Western blot detecting c-Myc protein level and loading control Actin in MLL-FP cells. One representative blot of three independent experiments is shown. **C.** ChIP-qPCR experiment detecting flag-tagged MLL-FPs or IgG on the *c-Myc* promoter and enhancer in MLL-FP cell lines. Error bars indicate standard deviation from ≥ 2 independent experiments. **D.** Relative viability of cells treated with JQ1 as detected by MTT assay. Data represents two independent experiments with four technical replicates. Statistics were analyzed by two-way ANOVA compared to MLL-AF9. *, *p* < 0.05; **, *p* < 0.01; ***, *p* < 0.001; ****, *p* < 0.0001. Calculated IC_50_ values are shown below. **E.** Western blot detecting c-Myc protein levels in cells treated with indicated dosage of JQ1.

To understand the regulation and role of c-Myc in MLL-FP leukemia cells, we performed qPCR to confirm differences in expression amongst MLL cell lines. Indeed, MLL-AF9, MLL-ENL and MLL-AF10 transformed cells expressed higher *c-Myc* transcripts than MLL-AF1p, MLL-Gas7 and MLL-AF6 (Figure 3A). Western blotting confirmed *c-Myc* overexpression translated to higher protein levels in MLL-AF9, MLL-AF10 and MLL-ENL compared to MLL-AF1p and MLL-GAS7 cells (Figure 3B). Interestingly, MLL-AF6 cells displayed Myc protein levels comparable to MLL-AF10, MLL-AF9 and MLL-ENL despite mRNA expression levels similar to MLL-AF1p and MLL-GAS7 (Figure 3A and 3B). It is noteworthy that MLL-AF6 transformation also resulted in the shortest disease latency in mouse leukemogenesis assays without a pronounced Myc signature (Figure 1C). Since phosphorylation of Myc through the ERK signaling pathway can enhance protein stability [28], we investigated activation of this pathway in our MLL-FP cell lines. Indeed, MLL-AF6 cells showed robust phosphorylation of ERK, which may account for increased Myc protein (Supplemental Figure 3A). Collectively, MLL-FPs with relatively higher c-Myc protein levels (MLL-AF9, MLL-ENL, MLL-AF6, MLL-AF10) induced a significantly shorter disease latency compared to MLL-FPs with lower c-Myc protein levels (MLL-AF1p and MLL-GAS7) (Supplemental Figure 3B). We asked whether differences in *c-Myc* expression were caused by differential binding of MLL-FPs to the *c-Myc* locus by performing ChIP-qPCR. Immunoprecipitation of flag tagged MLL-FPs showed that all the MLL-FPs were recruited to Myc promoter regions and to lesser extent Myc enhancer regions, which led to H3K4 and H3K79 methylation (Figure 3C, Supplemental Figure 3C). The intensity of MLL-FP binding and di-methylation at H3K4 and H3K79 did not correlate with expression levels suggesting these factors do not contribute to differences in *c-Myc* gene regulation in MLL-FPs cells (Figure 3A, 3C and Supplemental Figure 3C). To evaluate the importance of *c-Myc* expression, we treated MLL-FP cell lines with the BET inhibitor JQ1, which inhibits the proliferation of multiple forms of leukemia by disrupting BRD4 regulation of *c-Myc* [29-31] and other factors such as *Myb* and *Ets* proteins [32, 33]. Cell viability assays demonstrated that while all MLL-FP cell lines were sensitive to JQ1 treatment, MLL-AF1p and MLL-Gas7 displayed the greatest sensitivity to JQ1, (IC_50_ of 38.44nM and 37.89nM, respectively) (Figure 3D). MLL-AF6, MLL-AF9, MLL-AF10 and MLL-ENL cells displayed significantly reduced sensitivity to JQ1 (IC_50_ = 74.33nM, 59.13nM, 55.99nM, and 47.02nM respectively)(Figure 3D). Western blotting confirmed JQ1 treatment leads to dose-dependent reduction of Myc protein in MLL-FP cell lines (Figure 3E). Finally, we mined *MYC* expression levels from human patients [34, 35]. These data show significantly increased expression of *MYC* in AML (including the 11q23 subpopulation) compared to bone marrow from healthy patients (Supplemental Figure 3D). Further, clinical studies have demonstrated *MYC* expression is correlated with a poor prognosis in AML [36]. These data suggest the c-Myc transcriptional program is universally required for viability amongst the tested MLL-FP cell lines and that higher *c-Myc* expression confers decreased sensitivity to Brd4 inhibition.

**Figure 4:**
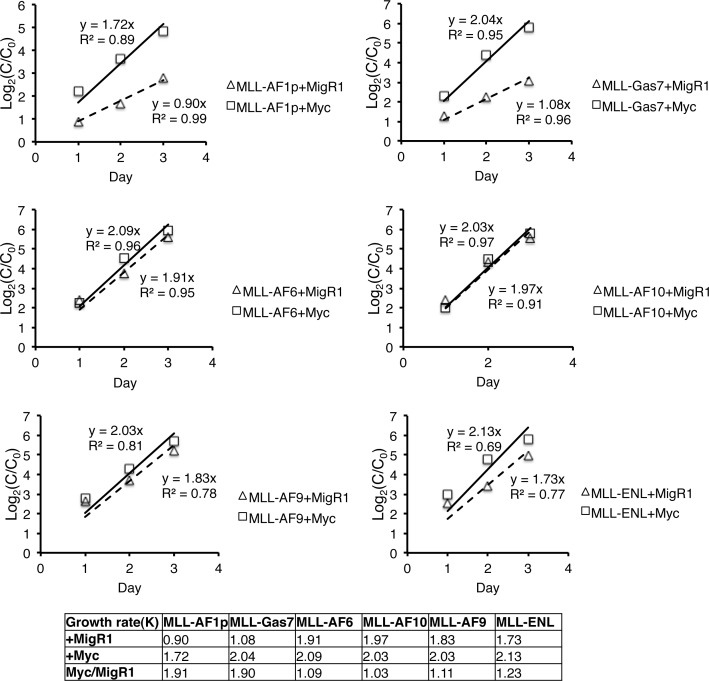
*c-Myc* expression increases the proliferative capacity of MLL-FP cells Growth rate of MLL-FP cell lines was determined following stable transduction of either empty MigR1 or MigR1-Myc. Data represents two independent experiments with technical duplicates. Only MLL-FP cells with lower c-Myc expression showed increased growth rates with overexpression of c-Myc. Differences in growth rates following overexpression of c-Myc in each MLL-FP cell line is shown relative to MigR1.

We next tested the functionality of *c-Myc* overexpression by establishing MLL-FP cell lines with and without stable *c-Myc* overexpression. An analysis of cooperation between *c-Myc* and MLL fusion proteins in vivo was precluded by induction of an aggressive leukemia with indistinguishable disease latencies by overexpression of *c-Myc* alone or in combination with MLL-AF9 or MLL-GAS7 (Supplemental Figure 4A) and previously reported [37]. Thus, MLL-AF1p, MLL-AF6, MLL-AF9, MLL-AF10, MLL-ENL and MLL-Gas7 cells were transduced with MigR1-Myc or MigR1 and stable GFP+ cells were collected for *in vitro* proliferation assays in liquid culture. The growth rate (K) was represented by the slope of the regressed line (Figure 4). Ectopic c-Myc expression had a minimal effect on MLL-FP cells that displayed increased levels of c-Myc protein including MLL-ENL, MLL-AF9, MLL-AF10 and MLL-AF6 (c-Myc(K)/MigR1(K) = 1.23, 1.11, 1.03 and 1.09 respectively) (Figure 4). In contrast, the proliferation rate of MLL-AF1p and MLL-GAS7 cells, which displayed relatively lower c-Myc protein levels, responded to overexpression with an ~ 2 fold increased growth rate (c-Myc(K)/MigR1(K) = 1.91 and 1.90 respectively) (Figure 4), Expression of ectopic Myc was similar or slightly reduced in MLL-AF1p and MLL-GAS7 compared to other MLL-FP cell lines confirming increased growth rates are not due to differences in ectopic Myc expression (Supplemental Figure 4). Collectively, these data suggest that c-Myc expression regulates proliferation rates of MLL leukemic cells.

### The c-Myc/Lin28/Let-7 pathway is differentially activated by MLL-FPs

**Figure 5:**
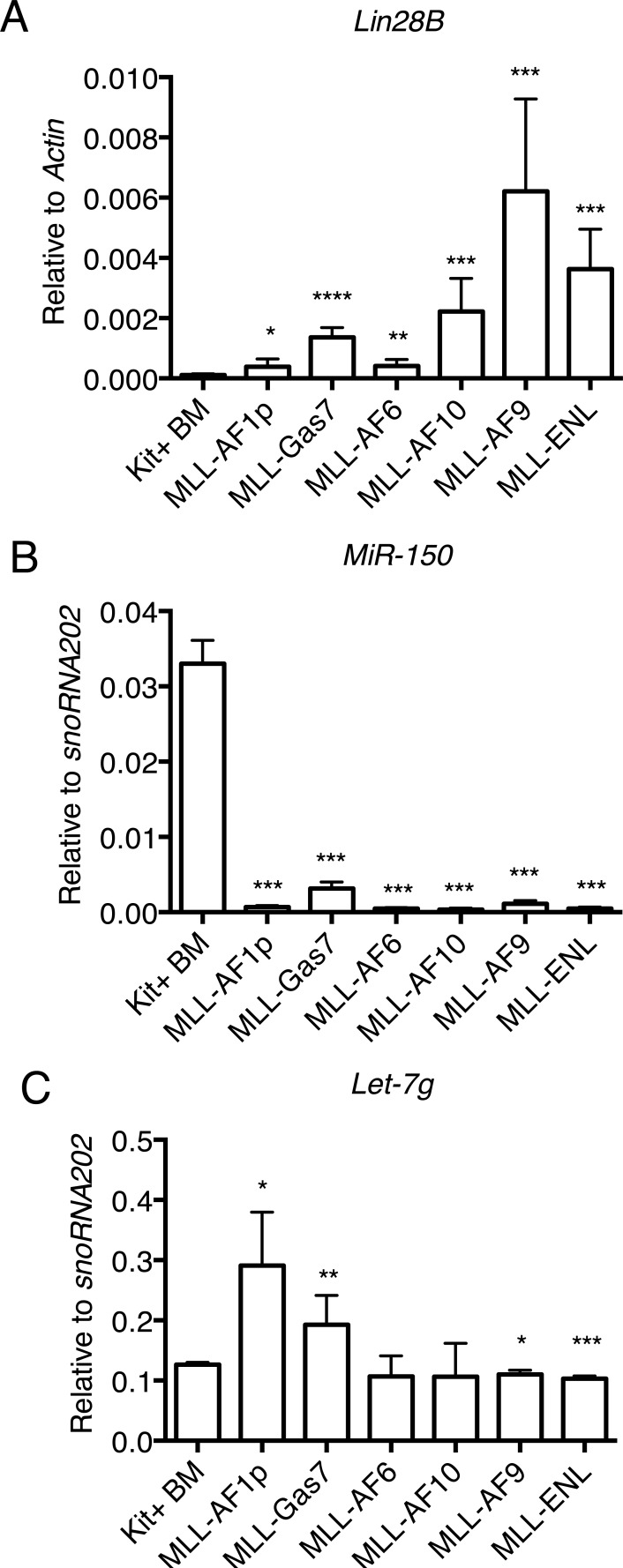
Lin28B and target miRNAs expression is altered in MLL-FP transformed leukemia cells **A.** Lin28B expression is increased in MLL-FP cell lines and reflective of *c-Myc* expression as assessed by qPCR. Errors bar indicate standard deviation from four independent experiments with technical duplicates. **B.** Mature miR-150 is repressed in all MLL-FP leukemia cells as detected by qPCR. **C.** let-7g expression is increased in MLL-FP cells with long disease latency as determined by qPCR. Error bars indicate standard deviation from two independent experiments with technical duplicates Statistics were done by *t*-test and were compared to kit+ bone marrow. *, *p* < 0.05; **, *p* < 0.01; ***, *p* < 0.001; ****, *p* < 0.0001.

Included in the deregulated c-Myc transcriptional program enriched in nuclear MLL-FP cells was the RNA binding and miRNA regulator *Lin28B* (Figure 2B and Supplemental Table 2). *Lin28B* is directly regulated by c-Myc and expression is associated with proliferation and invasiveness of multiple advanced human malignancies, including hepatocellular carcinoma, ovarian cancer, germ cell cancer and colorectal cancer [38-42]. Expression of *Lin28B* in c-kit+ mouse bone marrow cells was low (Figure 5A), consistent with previous reports [43]. Conversely, MLL-FP cells had significantly increased *Lin28B* expression compared to c-kit^+^ bone marrow cells with the highest expression in MLL-AF9, MLL-AF10, MLL-ENL reflecting *c-Myc* expression in these cells (Figure 5A). An important target of Lin28B in leukemic cells is miR-150 which negatively regulates c-Myc. *miR-150* maturation suppression by Lin28B is an important mechanism for MLL-FP mediated leukemic transformation [44]. Mature *miR-150* levels are reduced in all MLL-FP subtypes compared to c-Kit^+^ bone marrow cells consistent with previous findings that MLL suppresses *miR-150* expression through the Myc/Lin28B pathway (Figure 5B) [44]. Of note, *miR-150* expression was also significantly lower in AML-ETO9a and E2A-HLF leukemia cell lines compared to c-Kit^+^ bone marrow (Supplemental Figure 5A). We also noted that expression of the Lin28B target, *Hmga2*, was not altered in MLL-FP cells suggesting this pathway does not account for phenotypic differences (Supplemental Figure 5B). These data suggest another target may be responsible for differences in proliferation and disease latency associated with different MLL-FPs. Lin28B also directly binds and inhibits the processing of the miRNA let-7 which can also target *c-Myc* and other transcripts for degradation and is commonly repressed in human cancers [45-49]. Of the *let-7* family, *let-7a* and *let-7g* are decreased and *let-7b* increased in AML compared to CD34+ bone marrow [50]. We examined the expression of mature *let-7a, b, c,* and *g* in the different MLL-FP subtypes by Taqman microRNA assays and discovered the level of *let-7g* is increased specifically in MLL-AF1p and MLL-Gas7 cells compared to Kit+ bone marrow cells (Figure 5C). We observed either insignificant changes or varied expression that did not correlate with disease latency for *let-7a, b, c* between Kit+ bone marrow cells and MLL-FP leukemia cells (Supplemental Figure 5C, 5D, 5E). These data point to a potential role for *let-7g* in regulating cell proliferation and the aggressiveness of leukemic cells.

### *Let-7g* induces leukemia cell differentiation

**Figure 6:**
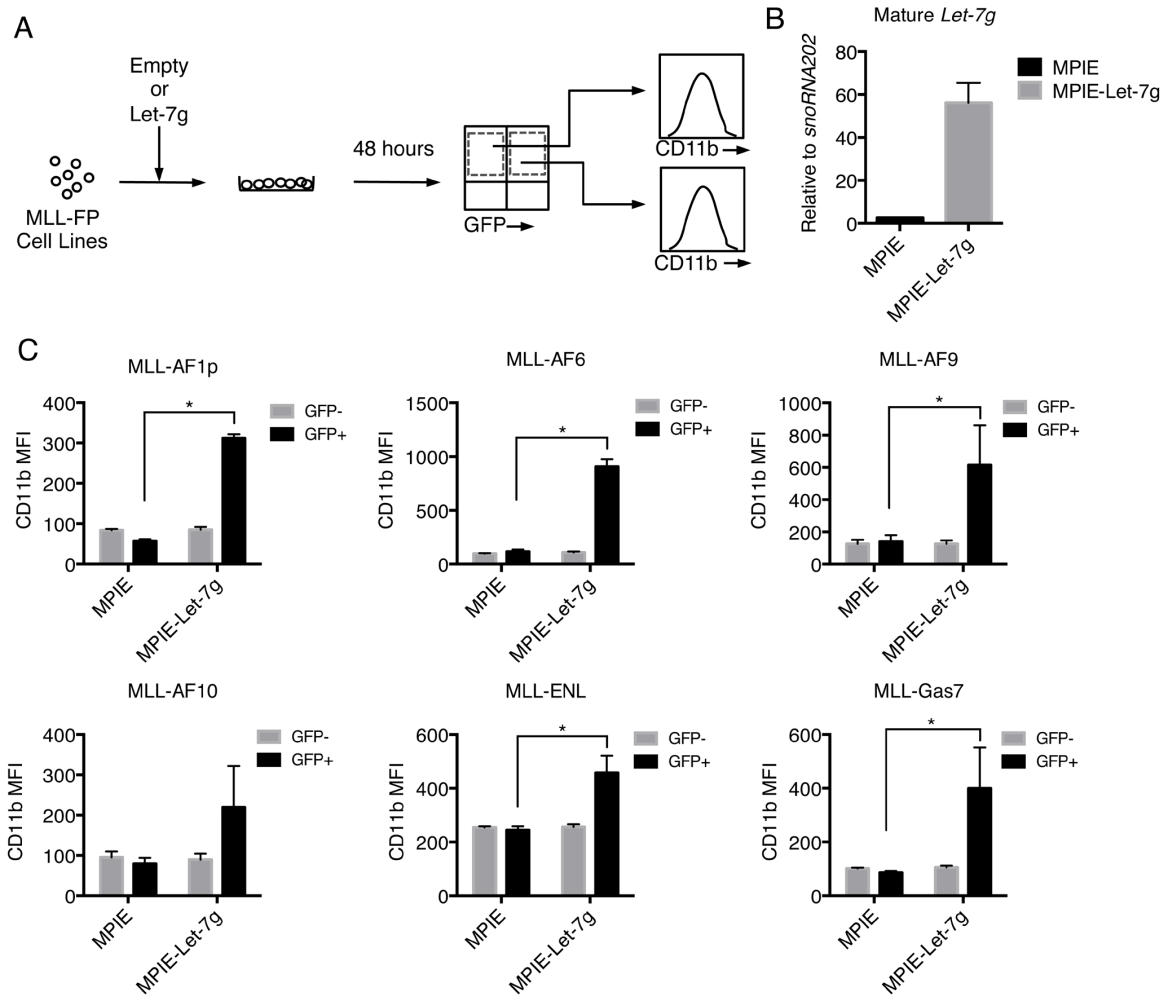
Overexpression of *let-7g* induces differentiation of MLL-FP leukemic cells **A.** Schematic of experimental protocol shows MLL-AF1p, MLL-AF6, MLL-AF9, MLL-AF10, MLL-ENL, and MLL-Gas7 cells were transduced with either MPIE or MPIE-let-7g. Cells were gated on GFP+ and GFP- populations and averaged expression of Cd11b on each population was calculated by mean fluorescence intensity (MFI). **B.** Increased expression level of mature *let-7g* in MPIE-let-7g transduced cells detected by miRNA qPCR. **C.** Increased Cd11b expression in MPIE-let-7g transduced MLL-FP cells compared to empty MPIE transduced cells. Error bars indicate standard deviation from two independent experiments with technical duplicates. Statistical significance determined by *t*-test. *, *p* < 0.05.

Decreased *let-7g* expression is associated with a poorer prognosis and increased metastasis of breast cancer while increased expression is associated with a more differentiated tumor phenotype [51]. Given the correlation between *Let-7g* and longer disease latencies observed in MLL-GAS7 and MLL-AF1p leukemias, we overexpressed *let-7g* in MLL-FP cells to test for increased differentiation using a miRNA retroviral vector (MPIE) that co-expresses GFP and examined the myeloid surface marker Cd11b (Figure 6A). Overexpression of mature *let-7g* was confirmed by qPCR (Figure 6B). MLL-FP cell lines were transduced with either MPIE or MPIE-*let-7g* retrovirus and cells were analyzed by flow cytometry to detect Cd11b and c-Kit expression (Figure 6A). GFP+ and GFP- cells expressed similar levels of Cd11b as shown by the mean fluorescence intensity (MFI) following transduction with empty MPIE (Figure 6C). Conversely, the GFP+ population displayed significantly increased Cd11b surface expression in MLL-AF1p, MLL-AF6, MLL-AF9, MLL-ENL and MLL-Gas7 cells following transduction with MPIE-*let-7g* (Figure 6C). The GFP- population served as an internal control and expressed similar levels of Cd11b to control cells (Figure 6A and 6C). In addition, MLL-AF6, MLL-AF9, MLL-AF10 and MLL-Gas7 cells transduced with *let-7g* displayed decreased c-Kit expression compared to the same cells transduced with vector control (Supplemental Figure 6). These data suggest increased expression of *let-7g*, as observed in MLL-AF1p and MLL-GAS7 cells, results in a more differentiated leukemic phenotype.

## DISCUSSION

MLL is rearranged with more than 100 partner genes in both pediatric and adult acute myeloid leukemia and generally associated with a poor prognosis. However, recent studies have revealed an association between the MLL fusion partner and disease latency [2, 3]. For example, the t(6;11) or t(10;11) translocations portend a poor prognosis with a 5-year event free survival (EFS) of less than 35% [2, 3]. Conversely, the t(1;11) translocation, coding MLL-AF1q, is associated with a good prognosis with an EFS of > 80%. Many patients with *MLL* translocations also harbor mutations in other genes, such as *FLT3* and *PTPN11*, which complicate the assignment of specific functions to each mutation [52, 53]. Here, we used *in vitro* and *in vivo* systems expressing various MLL-FPs to study the contribution of the MLL-translocation partner to transcriptional programs and disease latency. Our study demonstrates that different MLL fusion partners impart differential disease latency, which is positively correlated with *c-Myc* expression, a direct target of MLL-fusion proteins [54, 55]. Further, the miRNA regulator protein and c-Myc target gene, *Lin28B*, is increased in MLL leukemia cells compared to bone marrow. Both *miR-150* and *let-7* are repressed by both c-Myc and Lin28B [42]. We observed a striking repression of miR-150 in MLL-FP transformed leukemia cells in comparison to bone marrow. This is consistent with previous reports showing an association of Lin28B with malignant tumors and miR-150 repression in MLL leukemias [44]. However, modulation of another Lin28B target, *let-7g*, was most correlative with leukemia disease latency (Figure 5). Functionally, *let-7g* induces differentiation of MLL leukemic blasts suggesting this axis plays a role in blocking differentiation. Importantly, in addition to RAS, HMGA2 and cyclinD, *let-7* targets c-Myc for repression [56]. Thus, increased *c-Myc* expression positively feeds back on itself through increased Lin28B mediated repression of *let-7g*. Interestingly, *Let-7a* is reported to impart chemoresistance in AML cells bearing NPM mutations while increased *Let-7c* expression can promote granulocyte differentiation of PML/RARα leukemia cells [57, 58]. Our data demonstrates the importance of *Let-7g* in AML cells and is consistent with a model whereby MLL-FPs associated with short disease latency induce higher levels of *c-Myc* and *Lin28B* expression compared to MLL-FPs with longer disease latency. While *miR-150* is repressed uniformly across MLL subtypes, *let-7g* is differentially repressed by Lin28B according to expression level, which reinforces high c-Myc expression in MLL leukemias with short disease latency.

Our experiments suggest an exception to this model in the case of MLL-AF6. Despite low *c-Myc* transcription, protein was detected similar to MLL-AF9, MLL-ENL and MLL-AF10 (Figure 3A, 3B). Increased c-Myc protein correlates with a shorter disease latency (Figure 1C) but not high *Lin28B* expression (Figure 5A). The high Myc protein level in MLL-AF6 cells may result from increased Myc protein stability. MLL-AF6 activates the Ras pathway, which can protect Myc protein from ubiquitination-mediated degradation by modulating its phosphorylation at Thr58 and Ser62 [59, 60]. Indeed, we observed robust activation of Ras signaling pathway in MLL-AF6 cells (Supplemental Figure 3A). Thus, MLL-AF6 leukemias are likely initiating a unique gene program downstream of both Ras and MLL-AF6 that contributes to disease.

To account for differences in *c-Myc* expression, we examined MLL-FP binding and epigenetic regulation of the *c-Myc* locus. Interestingly, we detected similar binding of all MLL-FPs to the *c-Myc* gene promoter region, suggesting differential binding does not account for expression differences. We also observed similar H3K4 and H3K79 methylation on the *c-Myc* promoter region. The methylation level in MLL-FP cells does not correlate with transcript level, suggesting a decoupling of these histone marks with transcription of the *c-Myc* locus. Differential regulation of rates of transcription may account for changes in *c-Myc* expression, however further studies are needed to address this question.

BET inhibitors have gained much attention because of their potential therapeutic value in treating various forms of cancer including leukemia, breast cancer, prostate cancer and others [30, 31] [61, 62]. These inhibitors function, in part, through disruption of Brd4 mediated regulation of *c-Myc* expression. While all MLL-FP cells were sensitive to BET inhibition, our data shows that MLL-FPs associated with higher *c-Myc* expression are more resistant to BET inhibition (Figure 3D). This implies a general importance of the *c-Myc* transcriptional pathway in MLL leukemias and suggests that patients harboring MLL-FPs that induce higher levels of *c-Myc* expression will display increased resistance to BET inhibition.

## MATERIALS AND METHODS

### Mice

Female C57Bl/6 mice at 8-12 weeks old were purchased from Taconic Farms. Mice were inoculated with 300μl of 10mg/ml 5-Flurouracil (5-FU) 5 days before bone marrows were harvested for hematopoietic stem and progenitor cell (HSPC) enrichment. MLL-FP transduced HSPCs were tail-vein injected into lethally irradiated mouse to establish murine leukemia models. Moribund mice were euthanized and tissues were collected for histology staining by histology laboratories of University of Michigan *in vivo* animal core. All animal studies were approved by the University of Michigan Committee on Use and Care of Animals and Unit for Laboratory Medicine.

### Cells

Kit+ bone marrow cells from 5-FU primed mice were enriched by Hematopoietic Stem and Progenitor Cell Enrichment Kit (Stem Cell Technologies) and transduced with freshly packaged retroviruses expressing MLL-FPs. Cells were selected with 1mg/ml neomycin for 1 week with refreshed antibiotics every 3 days. Established mouse leukemia cell lines were maintained in IMDM+15%FBS (for mouse myeloid long-term cultures, Stem Cell Technologies)+1%Pen/Strep+10ng/ml recombinant murine IL3 (R&D Systems).

### RNA-seq

Independently acquired duplicate RNA samples were collected within one month of MLL-FP cell line generation and sequenced for analysis. Total RNA was purified by RNeasy plus mini kit (Qiagen) following manufacturer's instructions and treated with RNase-free DNase. High quality total RNA with OD260/OD280 = 2, OD260/OD230 > 1.6 was used for sequencing. The University of Michigan sequencing core performed library preparation and sequencing. Briefly, the RNA samples were processed by Illumina TruSeq mRNA Sample Prep v2 kit using Oligo(dT). Deep sequencing was performed on Illumina™ HiSeq2000, and raw RNA-seq data were processed using the Illumina software pipeline. Sequenced reads were aligned to mouse reference genome (mm10) using Bowtie and Tophat (version 2.0.3) [63]. Counts per million (cpm) values were generated using HTseq [64]. Gene expression patterns were clustered with K-means (K = 6) using Cluster 3.0 [65]. Uncentered Pearson correlation was used in similarity matrix. Clustering results was visualized using the Bioconductor package *gplots* (R package version 2.4) [66, 67]. Gene Set Enrichment Analysis (http://www.broadinstitute.org/gsea/index.jsp) was performed using RPKM (reads per kb per million) as input. Pathways from Molecular signatures database (MSigDB) were used. Titles of the pathways were modified for simplicity: Myc Targets: HALLMARK_MYC_TARGETS_V1. These pathways were generated based on total of 8 public datasets by MSigDB. To analyze the enriched gene sets in nuclear fusion leukemias *vs* cytoplasmic fusion leukemias, averaged CPMs of MLL-AF9 and MLL-ENL cells were divided by averaged CPMs of MLL-AF1p, MLL-GAS7 and MLL-AF6 cells and genes with > 1.5 fold change were analyzed by Toppfun [25].

The RNA-seq data was deposited to GEO database (GSE73457).

### Proliferation assays

50000 cells were seeded in 2ml media in 12-well non-tissue culture treated plates with 2ml fresh media supplied at day 2. Cells were enumerated for 3 days by trypan blue exclusion. Growth rates were calculated according to exponential growth equation:

$$\begin{array}{l} C=C_{0}*2^{kt},\text{ transformed to}\\ {\text{Log}}_{2}\left(\frac{C}{C_{0}}\right)=kt \end{array}$$

C—cell numbers at counting days

C_0_—cell number at day 0

k—growth rate

t—days after plating

Log_2_(C/C_0_) was calculated by the cell number and plotted against time. Linear regression was performed with lines forced to pass (0,0) point to reflect no change at time 0.

### MicroRNA *let-7g* overexpression

*let-7g* precursor sequence was cloned into a retroviral-vector designed for miRNA expression, MXW-PGK-IRES-EGFP (MPIE)[68]. Plasmids were transfected to plat-E cells by Fugene6 reagent to package retroviruses. MLL-FP cells were spinoculated with retroviral supernatant in the presence of 5μg/ml polybrene 48 hour and 72 hour post plat-E cell transfection. EGFP and cell surface markers were detected by flow cytometry 48 hours post first spinoculation. The antibodies used were APC-labeled anti-mouse CD11b (clone M1/70, BD pharmingen), and APC-eFluor780 labeled anti-mouse c-Kit (clone 2B8, eBioscience).

### Microscopy

H&E stained slides were viewed with Olympus BX41 microscope and pictures were taken by Olympus BX41 camera. The software used for taking the pictures was cellSens Entry.

### QPCR

For detecting mRNA expressions, RNA was extracted by trizol according to the manufacturer's instructions. First-strand cDNA was made by Superscript III first-strand synthesis system (Life technologies). Fast SYBR green master mixture was used for the quantitative polymerase chain reaction (QPCR). For detecting microRNA expression, RNA was extracted by miRNeasy mini kit (Qiagen), reverse-transcribed by Taqman microRNA reverse transcription kit and detected by Taqman microRNA assays (Life technologies). QPCR was performed on Applied Biosystems 7500 Real-time PCR system to detect the transcript level. Primers used were listed in Supplemental Table 3.

### Flow cytometry

Antibodies used for detecting the surface markers were PE labeled anti mouse c-Kit (clone 2B8), APC labeled anti mouse CD11b (clone M1/70). For cell cycle analysis, cells were fixed with 75% EtOH overnight at 4°C, 5μg/ml Propidium Iodide (Life technologies) was added to PBS-washed cells and incubated at room temperature at dark for 20min and samples were run on flow cytometer right after the incubation. Flow data was collected by LSRII flow cytometer (BD) and analyzed by Flowjo software (Treestar).

### Western

Cells were harvested and lysed by SDS-PAGE loading buffer and heated in the presence of 2-mercaptoethanol at 95°C for 7min. Samples were run on 4%-20% or 6% Tris-Glycine SDS-PAGE gels (Biorad). Proteins were transferred from SDS-PAGE gel to nitrocellulose membrane (life technologies) in transfer buffer containing 10% Methanol at 25V for 1 hour and 45min. Membranes were blocked with 5% milk, and incubated with primary antibody at 4°C overnight. Membranes were washes with TBS-T buffer and incubated with secondary antibody at room temperature for 1~2 hours and washes again. After washing, membranes were developed by Supersignal West Pico or Femto reagents (Thermo Scientific) as needed. Primary antibodies used were anti-Myc antibody (Cell Signaling Technologies, clone D84C12), anti-Actin antibody (Sigma, clone AC-74) and anti-MLL antibody (Upstate, clone 9-12).

### MTT assay

Cells were seeded in flat-bottom 96-well plate at 5000 cells/well in 100ul total media containing DMSO control or JQ1 at concentrations indicated in figures. At 48 hours post JQ1 treatment, 10ul of 5mg/ml MTT was added to each well and incubated with the cells for 4 hours at dark in 37°C incubator. After 4-hour incubation, 100ul of 10%SDS-0.01M HCl was added to the wells and the plate was incubated overnight at 37°C. The absorption at 570nm was read by Eon microplate reader (Biotek) with pathlength correction after the incubation. The blank-corrected 570nm reading was exported for data-analysis.

### Chromatin immunoprecipitation-QPCR (CHIP-QPCR)

30×10^6^ cells were harvested and fixed with 1% paraformaldehyde for 15min at room temperature. Cells were washed twice with cold DPBS, snap-frozen and stored in -80°C before experiments. Cell pellets were lysed and sonicated by Bioruptor to generate short chromatins with 200~500bp length. Cell lysate were spun at 4°C to remove any debris and extra SDS. Supernatant was diluted 10 times before immunoprecipitation. 1% of diluted samples were saved as input. 10% of diluted samples were incubated with antibodies at 4°C overnight. Magnetic protein G beads were incubated with the samples at 4°C for additional 1~2 hours before the beads were washed sequentially with low-salt, high-salt, LiCl, and TE buffer. Samples were eluted from magnetic beads by freshly made NaHCO3-SDS buffer and DNA-was de-crosslinked from proteins by high salt and incubating at 65°C overnight. DNA fragments were purified by Qiaquick PCR purification kit (Qiagen) and were quantified by QPCR. Primers for CHIP-QPCR are listed in Supplemental Table 3.

## SUPPLEMENTARY MATERIAL FIGURES AND TABLES




