# Supplementary material for: Differential regulation of the c-Myc/Lin28 axis discriminates subclasses of rearranged MLL leukemia
Source: Oncotarget. 2016 Mar 19;7(18):25208–23. doi: 10.18632/oncotarget.8199 (PMC5041898; doi:10.18632/oncotarget.8199)
Supplement: Supplementary file 1 [file oncotarget-07-25208-s001.pdf]

Differential regulation of the c-Myc/Lin28 axis discriminates subclasses of rearranged MLL leukemia

Supplementary Material

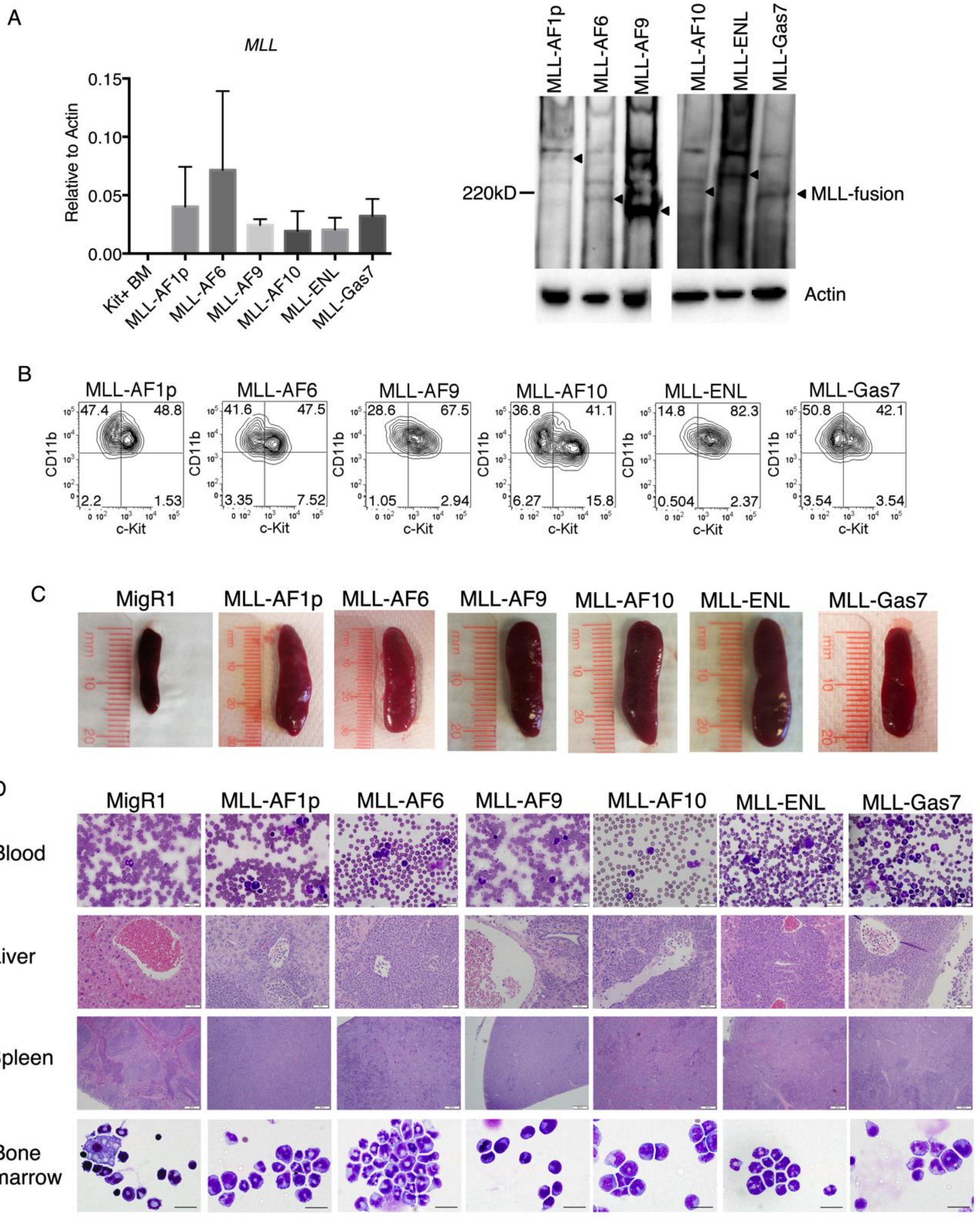

Supplemental Figure 1. (A-B) Expression of MLL-FP proteins in leukemia cell lines detected by QPCR and western blotting. Arrows indicate each MLL-FP protein. (C) Cd11b and c-Kit expression on MLL-FP transformed leukemia cells as detected by flow

cytometry. One of three independent experiments is shown. (D) Splenomegaly was observed in all diseased MLL-FP mice compared to control. (E) Histological analysis by H&E staining of liver and spleen, and Hema3 staining of peripheral blood and bone marrow shows increased blast cells of myeloid phenotype in MLL-FP mice and infiltration of leukemic blasts cells into liver and spleen. Scale bars represent 20μm for blood smear and bone marrow, 50μm for liver, and 200μm for spleen pictures.

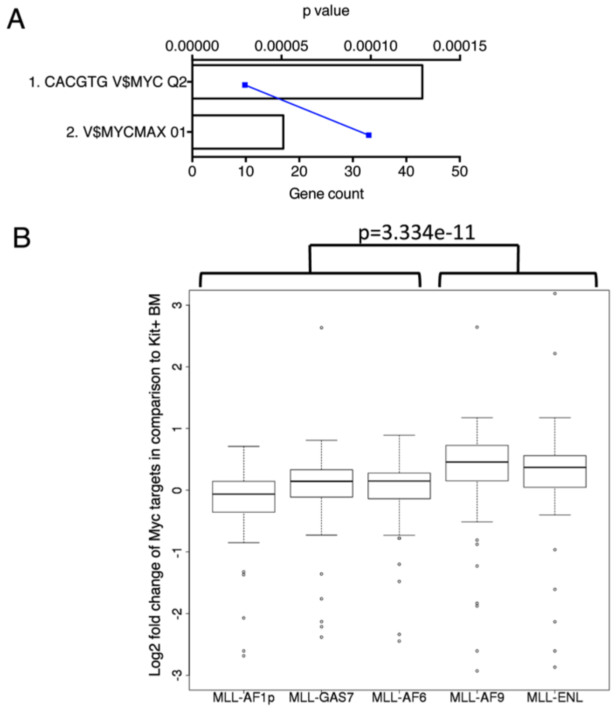

Supplemental Figure 2. (A) ToppGene analysis of transcription factor binding sites. Cluster 4 genes, described in Figure 2B, are enriched for Myc/Max binding sequences in MLL-AF9 and MLL-ENL cells compared to MLL-AF1p, MLL-GAS7 and MLL-AF6. Bars indicate gene count and blue points and line indicate p value. (B) Log2-fold change validated c-Myc target gene, as identified in Figure 3C, for each MLL-FP compared to bone marrow (Mann-Whitney U-test, p=3.3e-11). C-Myc target genes were the validated targets of C-MYC transcriptional activation in Figure 2C. **(C)**Expression level of genes previously identified as direct or indirect targets of MLL-FPs, as well as myeloid differentiation genes. Error bars indicate standard deviation of averaged RNA-seq counts per million data from duplicate samples.

C

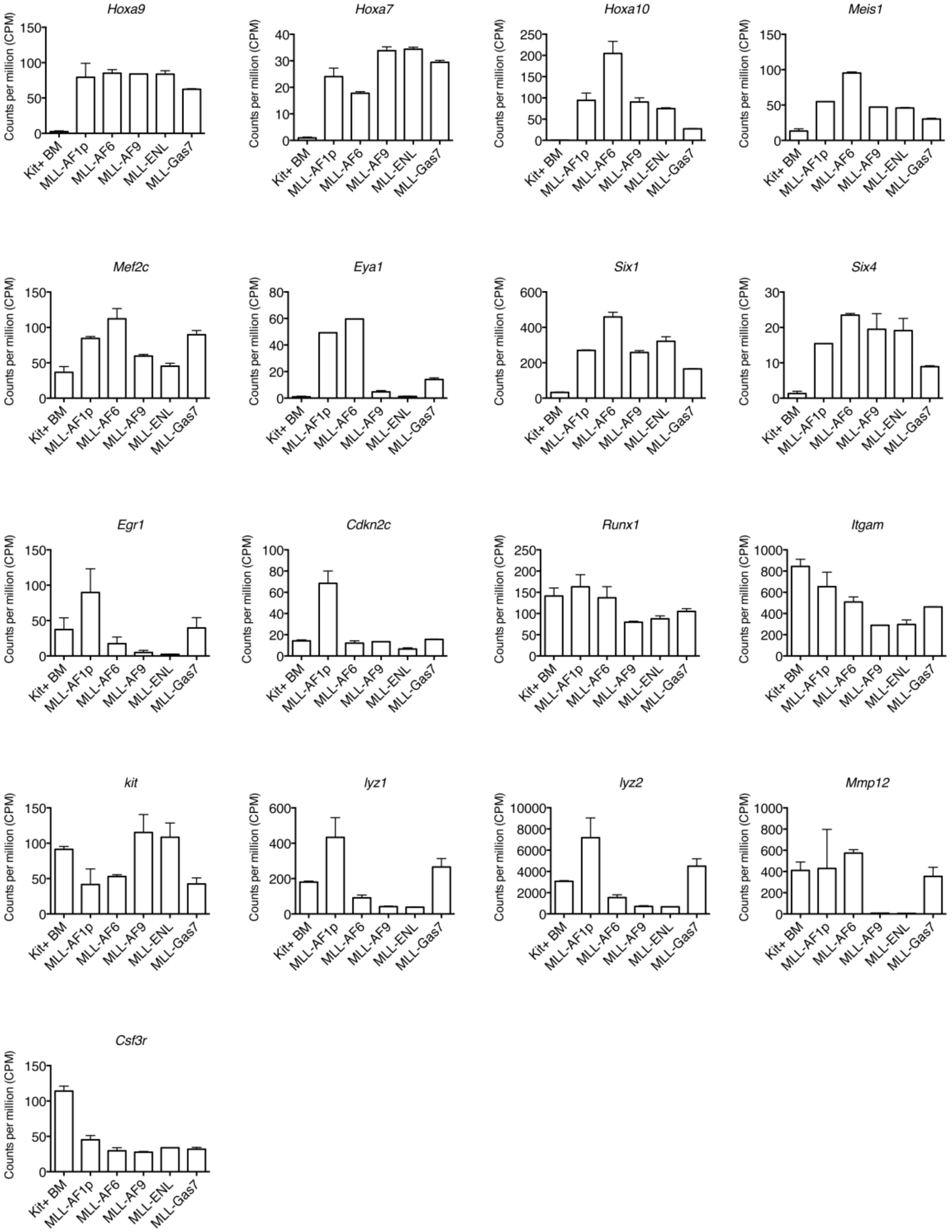
Hoxa9
Hoxa7
Hoxa10
Meis1
Mef2c
Eya1
Six1
Six4
Egr1
Cdkn2c
Runx1
Itgam
kit
lyz1
lyz2
Mmp12
Csf3r
Counts per million (CPM)
Kit+ BM
MLL-AF1p
MLL-AF6
MLL-AF9
MLL-ENL
MLL-Gas7

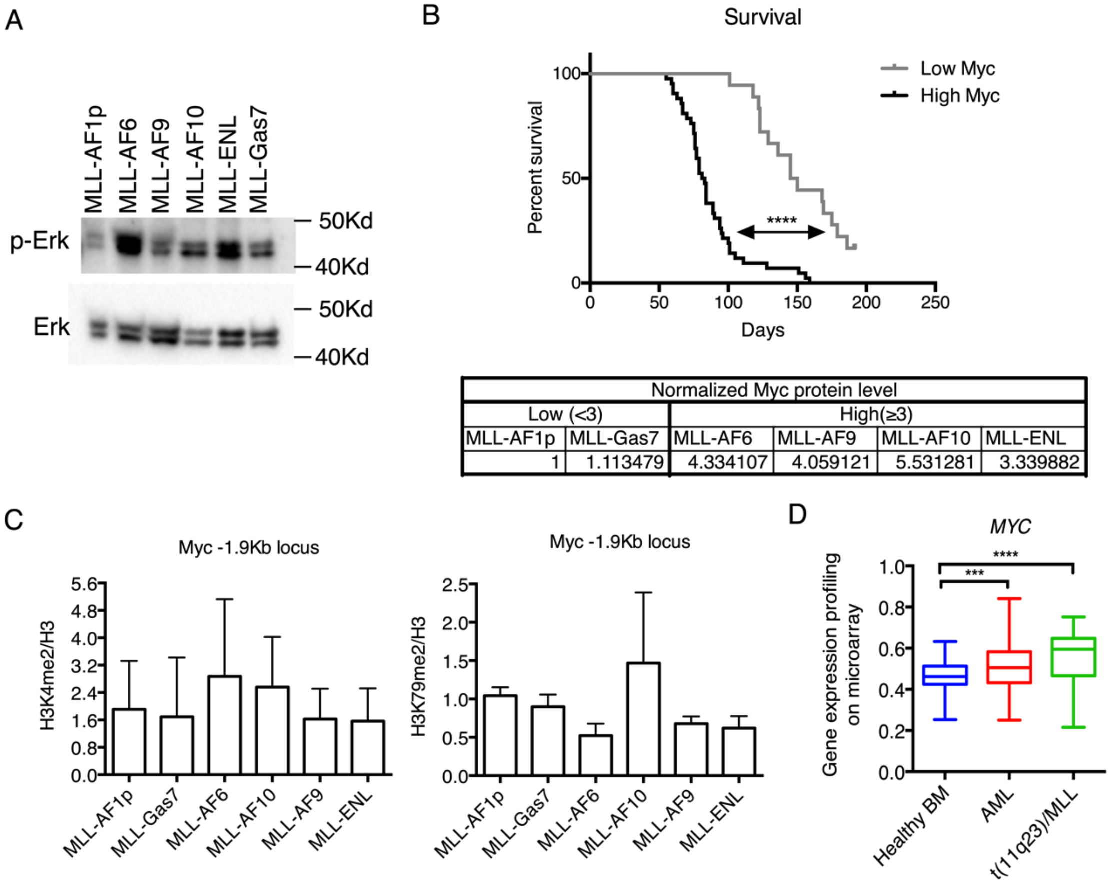

Normalized Myc protein level					
Low (<3)		High(≥3)			
MLL-AF1p	MLL-Gas7	MLL-AF6	MLL-AF9	MLL-AF10	MLL-ENL
1	1.113479	4.334107	4.059121	5.531281	3.339882

Supplemental Figure 3. (A) Western of Erk and phosphorylated Erk in MLL-FP cells. (B) Combined survival data for high and low expressing MLL-FP leukemias. High Myc MLL-FP leukemias include the MLL-AF9, MLL-ENL and MLL-AF6 group and the low Myc group include MLL-AF1p and MLL-GAS7 described in Figure 2. ****, $p<0.0001$ by Log-rank test. Myc protein level is calculated by densitometry and normalization to actin. Myc protein levels are shown relative to MLL-AF1p cells. (C) ChIP-qPCR for H3K4me2 and H3K79me2 histone marks on the *c-Myc* promoter in MLL-FP cell lines. Data shown relative to total H3. Error bars indicate standard deviation of three independent experiments. (D) MYC expression level in healthy control and AML and t(11q23)/MLL patients. Re-analyzed data from 2010 Haferlach et al. [34] downloaded from Leukemia Gene Atlas [33]. ***, $p<0.001$; ****, $p<0.0001$ by t-test.

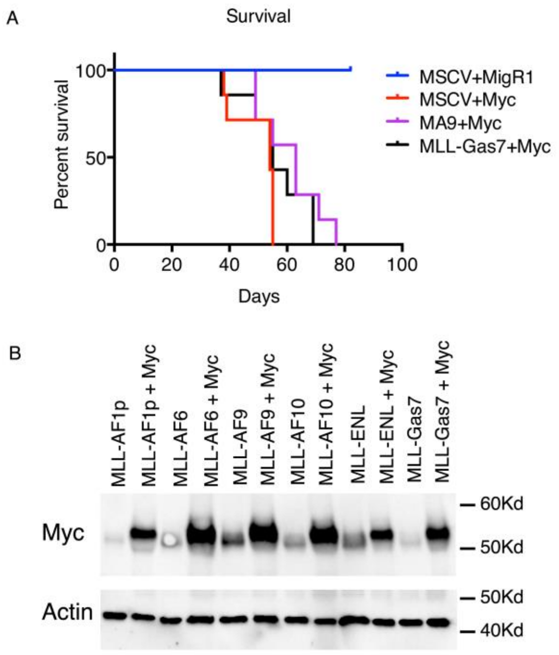

Supplemental Figure 4. (A) Survival curve of mice transplanted with indicated MLL-FP transduced cells with Myc co-expression or control. (B) Western blot showing Myc protein level in Myc overexpressed MLL-FP cells or control cells.

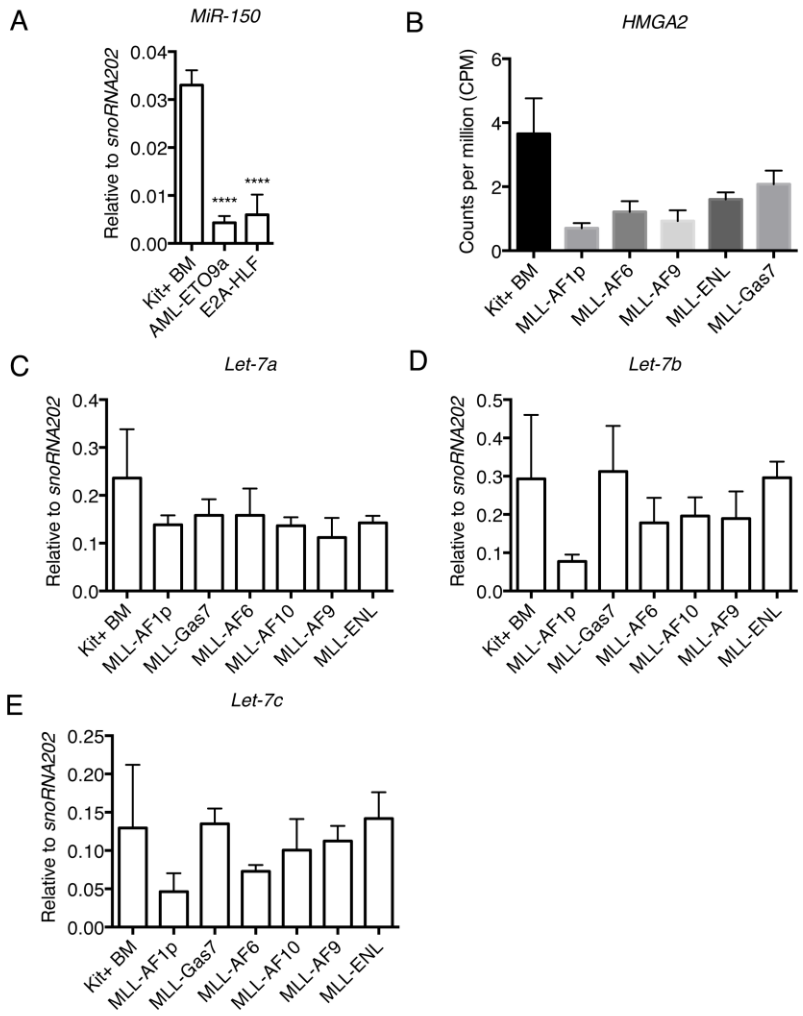

Supplemental Figure 5. (A) Mature miR-150 expression in lin$^-$c-kit$^+$ bone marrow cells, AML-ETO9a and E2A-HLF leukemic cells is shown as detected by qPCR. Error bars represent standard deviation of two independent experiments. ****, $p<0.0001$. (B) Hmga2 expression level detected by RNA-seq. (C-E) Mature let-7a,b,c levels are not significantly changed in MLL-FP cells compared to kit+ bone marrow cells. Error bars indicate standard deviation of two independent experiments.

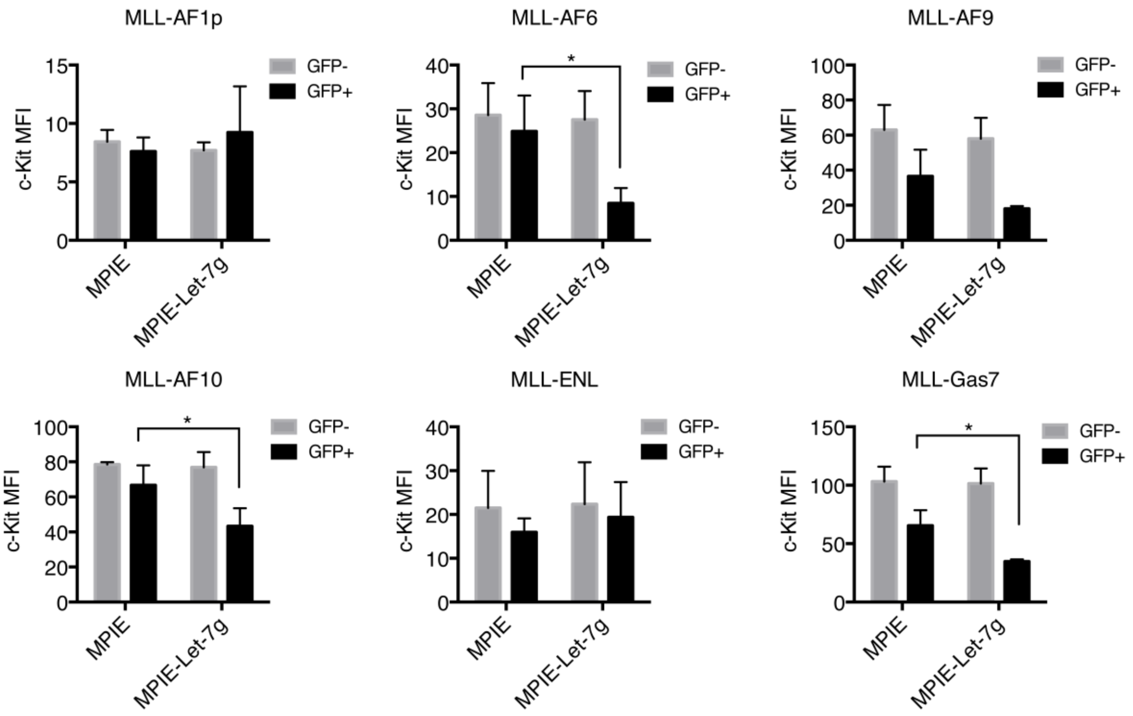

Supplemental Figure 6. *Let-7g* overexpression as described for Figure 7 was followed by flow cytometry detection of c-Kit expression in MLL-FP cells. MLL-AF6, MLL-AF9, MLL-AF10 and MLL-Gas7 cells show reduced c-Kit expression following overexpression of *let-7g*.

Supplemental Table 1 is submitted as a separate Excel sheet.

Supplemental Table 1. Expression of expressed genes in $lin^{-}c\text{-}kit^{+}$ bone marrow cells and MLL-FP cells. Log2 fold change is shown for each gene in MLL-FP cells in comparison to $lin^{-}c\text{-}kit^{+}$ bone marrow cells. In addition, gene expression, CPM, is shown for $lin^{-}c\text{-}kit^{+}$ bone marrow cells and MLL-FP cells. Data is shown for all genes and individualized clusters.

Supplemental Table 2. A list of genes identified by ToppGene as described for Figure 3C related to the c-Myc transcriptional pathway enriched in MLL-AF9 and MLL-ENL cells compared to MLL-AF1p, MLL-GAS7 and MLL-AF6. Also listed are genes identified by ToppGene described in Supplemental Figure 1 containing Myc or Max binding sequences.

Pathway	Validated targets of c-Myc transcriptional activation	Hmga1, Lin28B, Shmt1, Ruvbl1, Myc, Ncl, Ruvb2, Taf4b, Cad, Odc1
Transcription factor binding site	CACGTG V$MYC Q2	Hmga1, Fkbp11, Hmrnpa1, Hoxa4, Dlc1, Gar1, Xpo5, Cgn, Pla2g6, Zcchc7, Trim46, Ampd2, Set, Fmr1, Fpgs, Shmt1, Nr1d1, Akap12, Nop58, Igf2r, Stc2, Mthfd1, Gemin4, Myc, Pabpc4, 2700049A03Rik, Ncl, Nr6a1, Col25a1, Bcor, Mdn1, Ifrd2, Nfx1, Nol8, Satb2, Cad, Qtrtd1, Odc1, Zfp593, Tfap4, Atad3a, Cirh1a, Olfm2
	V$MYCMAX 01	HMGA1, FKBP11, HNRNPA1, TRIM46, AMPD2, NOP58, MTHFD1, KIAA0586, NCL, NR6A1, IFRD2, SATB2, CAD, QTRTD1, ODC1,TFAP4, ATAD3A

Supplemental Table 3. Primer sequences used in this study and let-7g precursor sequence cloned from C57BL/6 genome. All sequences are listed from 5’ end to 3’ end.

**QPCR primers**			
Species	Gene	Forward	Reverse
Mouse	Actin	GCCCTGAGGCTCTTTTCCAG	TGCCACAGGATTCCATACCC
Mouse	Lin-28B	CGGGTAACAGGCCCAGG	CGTCTCCACCTATCTCCCTTTG
Mouse	Myc	TCCTGTACCTCGTCCGATTC	TTGCTCTTCTTCAGAGTCGCT
**CHIP-QPCR primers**			
Species	Gene	Forward	Reverse
Mouse	Myc - 1.9Kb	ACAAATCCGAGAGCCACAAC	AACACCAAGAGCCACCAATC
**Cloned murine Let-7g sequence (C57Bl/6J, chromasome 9 nt 106178583 to 106179170)**			
ATGCCACCGATGCTTTTGGATAAGGATAAATACTAGAATTCTGAGGGTATATAAAAA<br>TATATATAAATGACTGGTGTATTTCTTTTGTTGGGTTGTTTTTCTAAGTGATTAAACAA<br>TTCTCCAAATATGGTAAAGATGAGGCAAATGTGTGGCGGGTGCAGCTTTGCTGCCT<br>GGCCTCTGCTGTGGGGATGTTGCCTTTCCTTCCTCAAGTGCGTCCTGCAGAGCTGC<br>TCCAGCGCTCCGTTCTCTTTTGCCTGATTCCAGGCTGAGGTAGTAGTTTGTACAGTT<br>TGAGGGTCTATGATACCACCCGGTACAGGAGATAACTGTACAGGCCACTGCCTTGC<br>CAGGAACAGTGCACCAGCTACCAAATGGGGTGGAGAAGATGGTGAAGCCCTGCTC<br>ATCTCTGGGATTCCAGGTAATGGGAGGGGATCTCTCTTTAGGTCAGGTGAGTAGTT<br>CCTGGCACTAGAATAACCAGAAATGCATTGTGGTTGTCATTTGAGAGGCTTGGAGT<br>TGTTCAGATAAACCGTTTGAAACTGTTACAGAATATATAGAAGGGTTCTTTTTTTCTC<br>TCTTATTGCAAATGCCCTCTCA			
